# A family study of congenital dysfibrinogenemia caused by a novel mutation in the FGA gene: A case report

**DOI:** 10.1515/med-2020-0214

**Published:** 2020-08-03

**Authors:** Yingli Qiao, Qisi Zhang, Poshi Xu, Yuhui Deng

**Affiliations:** Department of Laboratory Medicine, Henan Provincial People s Hospital, Department of Laboratory Medicine of Central China Fuwai Hospital, Central China Fuwai Hospital of Zhengzhou University, No. 1 Fuwai Road, Zhengzhou, Henan, 450003, China

**Keywords:** fibrinogen, congenital dysfibrinogenemia, gene mutation, case report

## Abstract

Congenital dysfibrinogenemia (CD) is a rare hereditary fibrinogen disorder characterized by normal fibrinogen antigen levels associated with lower functional activities. The aim of this study is to analyze the phenotype and genotype of a family of CD. Routine coagulation screening tests were performed on the proband, her parents, and her grandparents. Then, the purified genomic DNA extracted from peripheral blood was amplified by PCR, and Sanger sequencing was performed to further confirm the mutation. The prothrombin time and activated partial thromboplastin time of the proband were normal, thrombin time prolonged, and the activity of fibrinogen (Fg:Ac) decreased significantly, but fibrinogen antigen (Fg:Ag) level was normal. The coagulation function indices of the proband’s father and grandfather were similar to her, and the indices of her mother and grandmother were normal. Sequencing results showed that the proband had a heterozygous missense mutation in FGA gene c.92G > A, which caused the mutation of amino acid 31 from glycine to glutamic acid (p.Gly31Glu). Her father had the same heterozygous mutation. In conclusion, the proband suffered from CD. The change of Gly31Glu in A chain due to the c.92G > A heterozygous missense mutation in the FGA gene is the cause of CD in the family. To the best of our knowledge, the mutation site is new and first reported so far.

## 1 Introduction

Fibrinogen (Fg), a 340 kDa glycoprotein with coagulation function, is synthesized in the liver. It consists of three polypeptide chains: Aα, Bβ, and γ. It is encoded by three independent genes: FGA, FGB, and FGG, located in the region of 4q28-4q31 about 50 kb, and a symmetrical hexamer structure (AɑBβγ)_2_ is formed by several disulfide bonds [1,2]. Congenital dysfibrinogenemia (CD) is a rare hereditary fibrinogen disorder characterized by normal fibrinogen antigen levels associated with lower functional activities [3]. The majority of patients with CD is autosomal dominant inheritance. Molecular gene mutations of CD include base substitution, frameshift mutation caused by base insertion or deletion, small fragment insertion or deletion, cleavage site mutation, and regulatory region mutation, among which single base substitution is the most common. The majority of cases are due to heterozygous missense mutations in the coding region of one of the three fibrinogen genes (FGA, FGB, or FGG) [4,5]. There is a significant heterogeneity in the clinical manifestations of this disorder, including asymptomatic, bleeding, thrombosis, or both a bleeding tendency and thrombosis [6]. Gene mutation detection is the gold standard for the clinical diagnosis of CD. However, in the clinical diagnosis and treatment, it is necessary to combine the patient’s clinical manifestations, coagulation function indicators, and family status. In this study, we analyze the phenotype and genotype of a family of CD to further understand the pathogenesis of hereditary abnormal fibrinogenemia.

## 2 Case presentation

**Figure 1:**
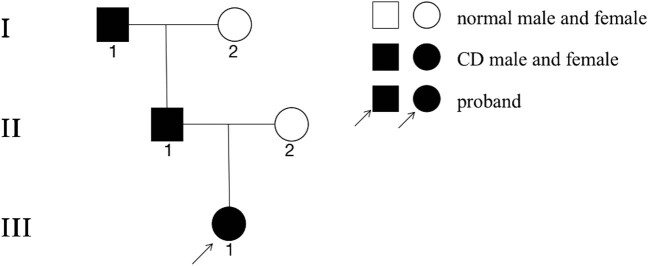
Pedigree of the family. The proband (III_1_) is indicated by the arrow.

The proband (III_1_, Figure 1), female, 14 month old, was admitted to the Children’s Cardiac Surgery Ward in the Central China Fuwai Hospital for “discovering the heart murmur of the child for more than half a year.” According to the chief complaint, medical history, physical signs, and cardiac color Doppler ultrasound, the clinical diagnosis was “congenital heart disease, atrial septal defect” with no family history of close relatives marriage and thrombotic diseases. Her father said that a family member of the previous generation had a history of nosebleeds for many years and the cause was unknown. Other family members had no history of bleeding.

**Statement:** All the family members have provided informed consent for publication of the case.

## 3 Diagnostic methods

### 3.1 Coagulation function test

Plasma was obtained from a venous blood vessel in the morning from the proband, her parents, and her grandparents, and it was anticoagulated by adding 0.109 mol/L sodium citrate in a 1:9 ratio. All specimens were centrifuged at 1,500*g* for 10 min to obtain platelet-poor plasma, and routine coagulation function tests including prothrombin time (PT), activated partial thromboplastin time (APTT), thrombin time (TT), antigen, and activity of fibrinogen within 2 h were performed. The activity of fibrinogen was measured by the Clauss method, while the antigen of fibrinogen was determined by immunoturbidimetry.

### 3.2 DNA isolation and Sanger sequencing

For genetic analysis, DNA from the proband and her parents was isolated from their peripheral blood using a DNA isolation kit (Qiagen; Hilden, Germany). Primers were designed according to GenBank sequences M64982, M64983, and M10014 of Fg, covering all exon regions and flanking sequences of the Fg gene. The total volume of PCR was 50 µL, including Taq PCR MasterMix of 25 µL, ddH_2_O of 18 µL, DNA template of 3 µL, and upstream and downstream primers of 2 µL, respectively. The reaction conditions were 94°C pre-denaturation for 5 min, 94°C denaturation for 30 s, 56–60°C renaturation for 30 s, 72°C extension for 1 min, a total of 30 cycles, and 72°C extension for 10 min. The PCR products were analyzed by agarose gel electrophoresis and tapping and then sequenced by Sanger sequencing (ABI 3730XL Applera Company America). Chromas software was used to blast the sequence of coding exon corresponding to NC-000004.12 (FGA, FGB, and FGG) gene published by the NCBI GenBank. The sequence acquired by reverse sequencing needed reverse complementarity and then compared with find the mutation type and locus.

## 4 Results

### 4.1 Coagulation function results

The routine coagulation test of the proband showed a normal PT of 11.7 s, a normal APTT of 34.7 s, but a longer TT of 30.5 s and a lower fibrinogen activity of 0.77 g/L (tested by the Clauss method). The fibrinogen antigen level tested by immunoturbidimetry was 1.79 g/L. The test results of all the family members are listed in Table 1.

### 4.2 Genetic analysis results

**Figure 2:**
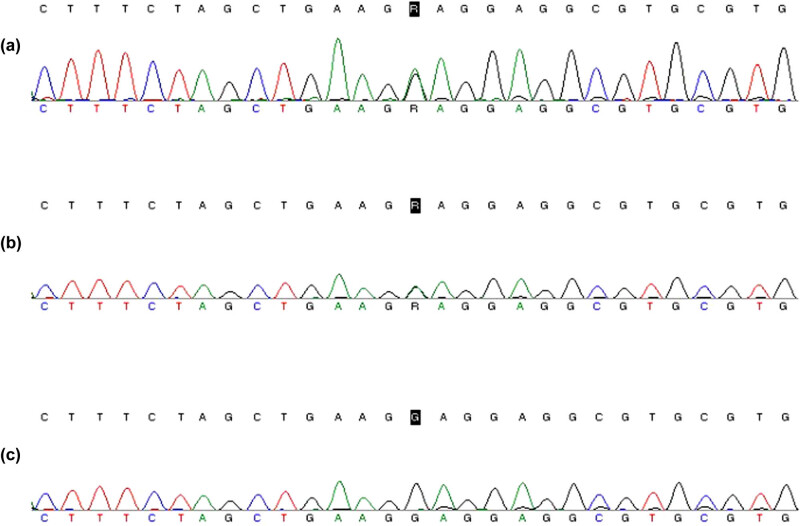
Sanger Sequencing Peak Map. (a) Proband (III_1_) FGA gene sequencing results; (b) proband’s father (II_1_) FGA gene sequencing results; and (c) proband’s mother (II_2_) FGA gene sequencing results.

Sequencing results showed that the proband had a heterozygous missense mutation in the FGA gene c.92G > A (Figure 2a), which resulted in the mutation of amino acid 31 from glycine to glutamic acid (p.Gly31Glu). Her father had the same heterozygous mutation (Figure 2b). There was no mutation in her mother’s fibrinogen gene (Figure 2c).

## 5 Discussion

In this study, the coagulation function test of the proband showed that TT prolonged and the activity of fibrinogen decreased significantly, while the antigen of fibrinogen was almost normal (Table 1). The routine blood coagulation test results of the proband’s father and grandfather were similar to her, and her mother’s and grandmother’s results were normal. Therefore, the proband was initially diagnosed as CD, which suggests a risk of surgical bleeding or thrombosis. The proband underwent transcatheter closure of atrial septal defect and repair of atrial septal defect with the continuous attention of surgeons. No significant bleeding or thrombosis occurred. Sequencing results showed that the proband had a heterozygous missense mutation in the FGA gene c.92G > A (Figure 2a). Her father had the same heterozygous mutation (Figure 2b). The genetic analysis results were consistent with the coagulation phenotype and further confirmed the diagnosis of CD of the proband.

**Table 1:** Coagulation function test results

Subjects	PT (s)	APTT (s)	TT (s)	Fg (g/L)		
				Fg:Ac	Fg:Ag	Fg:Ac/Fg:Ag
Proband	11.7	34.7	30.5	0.77	1.79	0.43
Proband’s mother	10.5	27.0	19.1	2.44	2.47	0.99
Proband’s father	11.3	24.8	30.7	1.10	2.52	0.44
Proband’s grandmother	9.5	28.9	19.6	2.48	2.23	1.11
Proband’s grandfather	11.9	23.1	30.9	1.07	2.14	0.50

PT, prothrombin time; APTT, activated partial thromboplastin time; TT, thrombin time; Fg, fibrinogen; Fg:Ac, fibrinogen activity; Fg:Ac, fibrinogen antigen.

In this study, the same heterozygous missense mutation in the FGA gene c.92G > A was found in both the proband and her father, which resulted in the mutation of amino acid 31 of the protein from glycine to glutamic acid (p.Gly31Glu). And we failed to find any information about the mutation in databases of fibrinogen mutation database, HGMD, gnomAD, ExAC, and 1000G databases [7,8,9,10,11]. It is a newly discovered mutation site. At present, the mechanism of this mutation affecting the activity of fibrinogen is not clear, which needs to be further explored. In this study, neither the proband nor her father had any clinical manifestation of hemorrhage or thrombosis. The proband’s father said that a family member of the previous generation had a history of nosebleeds for many years. Therefore, it cannot be excluded that the mutation may cause bleeding symptoms.

The clinical manifestations of patients with CD are heterogeneous, ranging from asymptomatic to bleeding and/or thrombosis [6]. The heterogeneity is mainly due to the diversity of molecular mechanisms. Previous studies have shown that about 40% of patients with CD have no symptoms, 40–50% of patients have a hemorrhage, 10–15% of patients have thrombotic diseases, and a small number of patients also have hemorrhage or thrombotic events [12]. A follow-up study conducted by Casini et al. found that some asymptomatic patients with CD at initial diagnosis still had hemorrhage or thrombosis events several years later, mostly in surgery, postpartum, or trauma status [13]. In this study, the proband is asymptomatic at present, but we could not estimate the risk of bleeding or thrombosis in the future. Consequently, the proband and her family still need accurate and regular follow-up and observation and treatment advice when necessary. Any treatment of patients with CD should be tailored to the individual and familial history. Asymptomatic patients with no history of bleeding or thrombosis need no special treatment except for close monitoring. Fresh frozen plasma, cryoprecipitate, or fibrinogen concentrate may be transfused when CD individuals or families have bleeding symptoms, of which fibrinogen concentrate is the first choice. Low molecular weight heparin can be used to prevent or treat thrombosis when thrombosis occurs.

## 6 Conclusion

In summary, we confirmed the diagnosis of CD of the proband by analyzing the phenotype and genotype of her family in this study. Both the proband and her father have detected the same heterozygous missense mutation in the FGA gene c.92G > A (p.Gly31Glu), which is new and first reported so far to our knowledge. The novel fibrinogen variant c.92G > A (p.Gly31Glu) was named fibrinogen Zhengzhou, and further studies in vitro could help us identify the definite molecular pathogenesis of this mutation.
